# Medicated Foley Catheters Do Not Prevent Catheter-Associated Urinary Tract Infection: A Systematic Review of Randomized Controlled Trials

**DOI:** 10.7759/cureus.66235

**Published:** 2024-08-05

**Authors:** Indraneel Banerjee, Jared Robinson, Indrajit Banerjee

**Affiliations:** 1 Department of Urology and Robotic Surgery, Penn Highlands Healthcare, Dubois, USA; 2 Department of Surgery, Sir Seewosagur Ramgoolam Medical College, Belle Rive, MUS; 3 Department of Pharmacology, Sir Seewosagur Ramgoolam Medical College, Belle Rive, MUS

**Keywords:** catheter-associated urinary tract infections (cauti), randomized control trials, surgery general, urinary catheters risks, catheter-related infections, urology, urinary catheter, prevention and control, urinary catheterization, urinary tract infections

## Abstract

Infections of the urinary tract are among some of the most common infections treated in clinical practice. Numerous risk factors play an intrinsic role in the development of such infections, namely: age, sexual intercourse, prolonged use of feminine hygiene products, instrumentation, pregnancy, sexually transmitted infections, obstructive uropathy such as prostatic enlargement or urethral strictures, compromised immunity, and constipation. A major cause of urinary tract infections (UTIs) in hospitalized patients is catheter-associated urinary tract infections (CAUTIs). This systematic review aims to identify the causative agents and risk factors and to determine whether nitrofurazone, silver alloy, or zinc oxide-impregnated or coated/medicated Foley catheters, or non-medicated (standard) Foley catheters, can reduce the incidence of CAUTIs.

A systematic review was conducted on the following databases: PubMed, Cochrane Central Register of Controlled Trials (CENTRAL), Trip medical database, and Google Scholar. A combination of keywords and Boolean operators was used ((((urinary tract infections) OR (urinary catheterization)) OR (prevention AND control)) ) AND (catheter-associated infections) for data extraction. All the randomized controlled clinical trials (RCTs) completed and available between January 1, 2005, and June 30, 2024, which focused on the prevention of CAUTIs, were screened thoroughly and were included in this systematic review. The Cochrane risk-of-bias tool for randomized trials (RoB 2) tool was used for risk of bias assessment. The Robvis visualization tool (McGuinness, LA, Higgins, JPT. Risk-of-bias VISualization (robvis): An R package and Shiny web app for visualizing risk-of-bias assessments. Res Syn Meth. 2020; 1-7) was used for development of traffic light plots and weighted bar plots for risk of bias. The literature search conducted produced 41,909 articles. Among these 19,076 were noted as duplicates and were excluded in the initial analysis; 22,833 manuscripts were thus screened after deduplication. Abstracts, case studies, reports, editorials, viewpoints, cross-sectional studies, cohort studies, case-control studies, case series, and letters to the editor/correspondence manuscripts (n = 22,745) were additionally excluded. A total of 88 full-text articles were assessed for eligibility. An in-depth evaluation and analysis further excluded 82 articles from the analysis quality assessment based on inclusion and exclusion criteria. Six RCTs were finally assessed regarding the prevention of CAUTIs and were ultimately included in the systematic review.

The primary causative agents involved in the CAUTIs were found to be mainly Gram-negative bacteria such as *Escherichia coli*, *Pseudomonas aeruginosa*, and *Enterococcus faecalis*. The risk factors noted for the development of these CAUTIs ranged from urethral trauma, overdistention of the bladder, prolonged catheterization, to improper handling of the urine bag. No significant advantage was noted between the use of medicated and non-medicated standard Foley catheters. The aseptic technique and indications followed for the catheterization play a vital role in the prevention of CAUTIs, and more cognizance thereof will aid in the reduction of the development of CAUTIs.

## Introduction and background

Urinary tract infections (UTIs) are among the most common infections treated in clinical practice, with the majority of these infections requiring antibiotics in order to subside [1-3]. Numerous risk factors play an intrinsic role in the development of such infections, including age, sexual intercourse, prolonged use of feminine hygiene products, instrumentation, pregnancy, sexually transmitted infections, obstructive uropathy such as prostatic enlargement or urethral strictures, compromised immunity, and constipation [4-6]. A major cause of UTIs in already hospitalized patients is catheter-associated urinary tract infections (CAUTIs), which are urinary tract infections contracted as a direct or indirect result of urinary catheterization [7-9]. These infections can be either simple or complicated and may result in severe outcomes, such as septicemia or even death [10]. The use of indwelling urinary catheters is increasing in both acute and chronic patient care. It is thus vital to understand the microbiology of the causative organisms and the methods by which these hospital-related infections can be minimized [11-14]. This systematic review aims to identify the causative agents and risk factors and to determine whether nitrofurazone, silver alloy, or zinc oxide-impregnated or coated/medicated Foley catheters, or non-medicated (standard) Foley catheters, can reduce the incidence of CAUTIs.

## Review

Methodology

Preferred Reporting Items for Systematic Reviews and Meta-Analyses (PRISMA) 2020 guidelines were implemented during the conduction of this systematic review.

Literature searches

An extensive review of the literature was done on the following databases: Pubmed, Cochrane Central Register of Controlled Trials (CENTRAL), Google Scholar, and Trip medical database (Table 1). 

**Table 1:** Various databases searched; Boolean operators and keywords used

Databases searched	Boolean operators and keywords	Total number of articles
PubMed	((((urinary tract infections) OR (urinary catheterization)) OR (prevention AND control)) ) AND (catheter-associated infections); Filters: from 2005 - 2024	6,433
Cochrane Central Register of Controlled Trials (CENTRAL)	urinary tract infections OR urinary catheterization OR prevention AND control AND catheter-associated infections; Filters: custom range 01/01/2005- 30/06/2024	8,304
Google Scholar	urinary tract infections OR Urinary Catheterization OR Prevention and Control AND catheter-associated infections; Custom range: 2005 - 2024	18,600
Trip	(((urinary tract infections) OR (urinary catheterization)) OR (prevention AND control)) AND (catheter-associated infections; Filter: from 2005 - 2024	8,572
		41,909

A combination of keywords was used for data extraction (((urinary tract infections) OR (Urinary Catheterization)) OR (Prevention and Control)) AND (catheter-associated infections). The following Medical Subject Headings (MeSH) term combinations were used "urinary tract infections" OR "urinary" AND "tract" AND "infections" OR "urinary tract infections" OR "urinary catheterisation" OR "urinary catheterization "OR "urinary" AND "catheterization" OR "urinary catheterization" OR "prevention and control" OR "prevention" AND "control" OR "prevention and control" AND "catheter-related infections" OR "catheter-related" AND "infections" OR "catheter-related infections" OR "catheter" AND "associated" AND "infections" OR "catheter-associated infections".

Inclusion criteria

All the randomized controlled clinical trials (RCTs) completed and available between January 01, 2005, and June 30, 2024, which focused on the prevention of CAUTIs were screened thoroughly and were included in this systematic review. Full-text RCTs published in English were identified and incorporated in this systematic review. 

Exclusion criteria

The exclusion of a trial was subject to the availability of the data concerning the prevention of CAUTIs. Non-randomized clinical trials, cohort studies, case-control studies, cross-sectional studies, abstracts, case studies, reports, editorials, viewpoints, case series, and letters to the editor/correspondence manuscripts were rejected from this systematic review.

Data extraction

Data extraction was conducted on the relative titles. The titles were initially examined based on their abstracts. Thereafter, the full texts of the examined titles of the RCTs that met the eligibility requirements were considered for the final selection. The literature evaluation was separately performed by IB, JR, and IB. The extracted data included study authors, year, design, sample size, study population, control, bacteriuria with urine colonies greater than 105 colony-forming units (CFU) per milliliter, microbiological culture, intervention, UTI incidence, the main findings, limitations of the study, and the study outcome.

Risk of bias assessment

The Cochrane risk-of-bias tool for randomized trials (RoB 2) was used for risk of bias assessment. The RoB 2 tool is best suited and implemented to assess the methodological quality of randomized controlled trials. The data were transferred into the Robvis visualization tool (McGuinness, LA, Higgins, JPT. Risk-of-bias VISualization (robvis): An R package and Shiny web app for visualizing risk-of-bias assessments. Res Syn Meth. 2020; 1-7) for development of traffic light plots and weighted bar plots for risk of bias summary and figure. 

Results 

**Figure 1:**
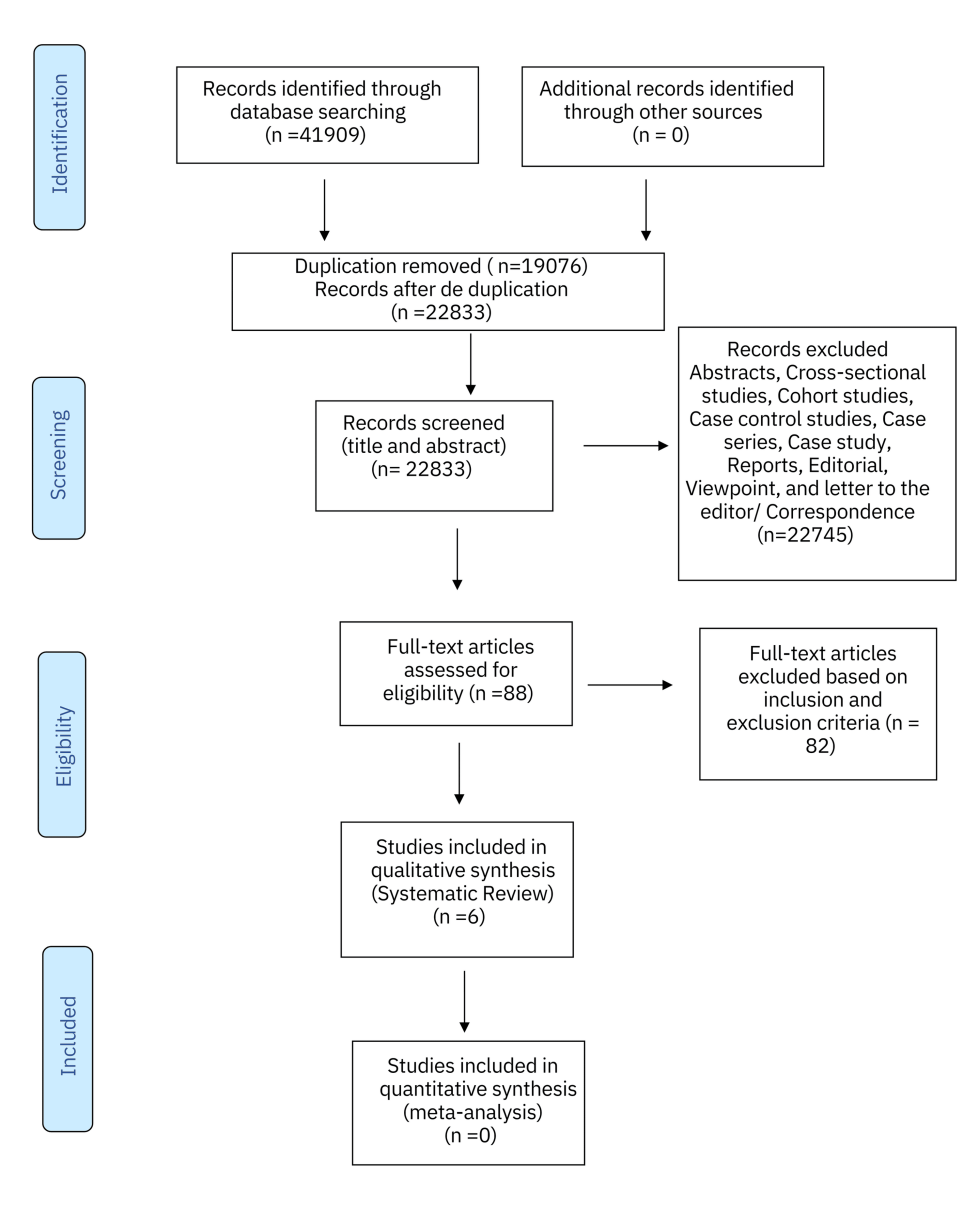
A PRISMA flowchart outlining the study selection process for the systematic review PRISMA: Preferred Reporting Items for Systematic Reviews and Meta-Analyses

The literature search conducted produced 41,909 articles. Among these, 19,076 were noted as duplicates and excluded from the initial analysis. Thus, 22,833 manuscripts were screened after deduplication. Abstracts, case studies, reports, editorials, viewpoints, cross-sectional studies, cohort studies, case-control studies, case series, and letters to the editor/correspondence manuscripts (n = 22,745) were additionally excluded. A total of 88 full-text articles were assessed for eligibility. An in-depth evaluation and analysis further excluded 82 articles from the analysis quality assessment based on inclusion and exclusion criteria. Six RCTs were finally assessed regarding the prevention of CAUTIs and were ultimately included in the systematic review (Figure 1).

**Figure 2:**
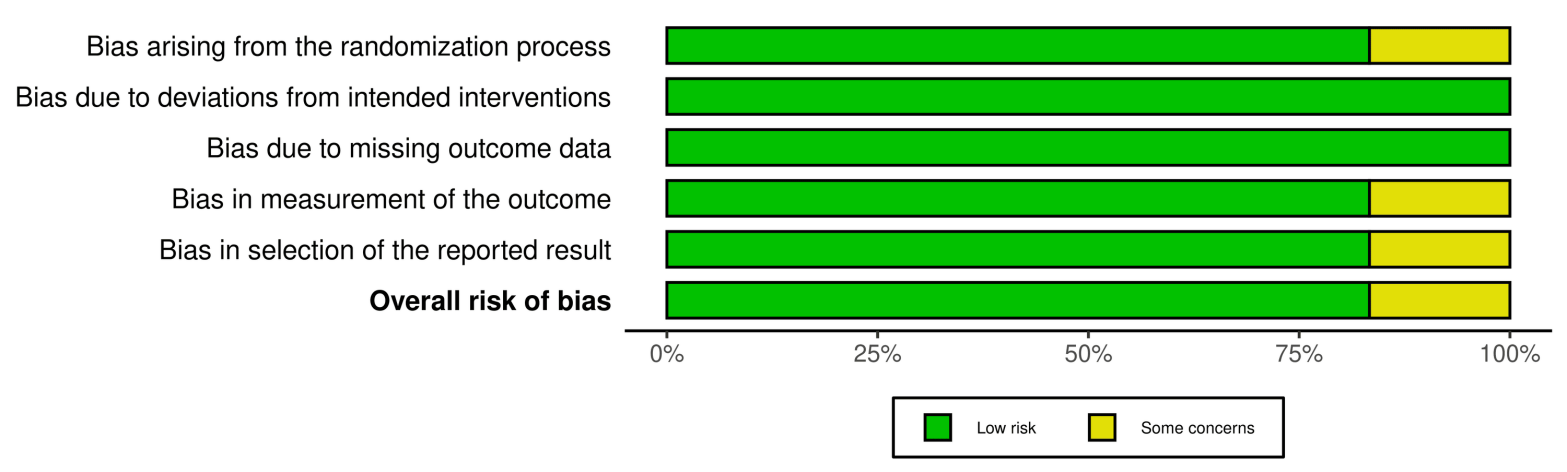
Summary of the risk of bias for RCTs (weighted bar plots) RCTs: randomized controlled clinical trials

**Figure 3:**
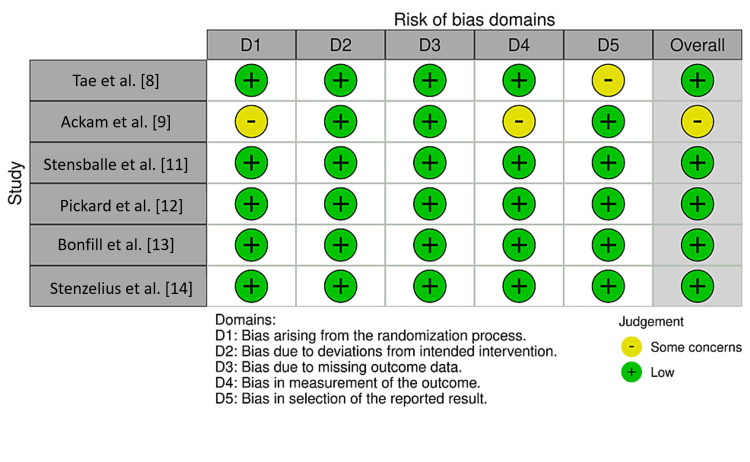
Figure of risk of bias of RCTs (traffic light plot) RCTs: randomized controlled trials

Figures 2-3 depict the risk of bias assessment based on the RoB 2 tool. The Robvis visualization tool, a web-based application program, was used for the development of traffic light plots and weighted bar plots for risk of bias summary and figure. Figure 2 shows the weighted bar plots for the risk of bias summary. Figure 3 shows a traffic light plot. The figure of the risk of bias was generated based on five domains. All the included RCTs underwent a quality assessment by the RoB 2 tool, which showed good overall results of low risk of bias in the randomization process (low risk 83.3%), deviations from intended interventions (100% low risk), missing outcome data (100% low risk), measurement of the outcome (low risk 83.3%), and selection of the reported result (low risk 83.3%), and overall risk of bias for the six RCTs were found to be low risk (83.3%) and 16.7%, which signified some concerns.

Tables 2-3 depict the country of study, the number of intervention patients, the number of control group patients, the study design, inclusion criteria, the microbiological culture, the combination of interventions prescribed, the UTI incidence, and the outcomes of the interventions.

**Table 2:** Country of study, duration of catheter use, design, sample size, and inclusion criteria CAUTI: catheter-associated urinary tract infection; CFU: colony-forming units

Author, year	Country	Duration of catheter use	Design	Intervention patients	Control	Inclusion criteria
Tae et al., 2022 [8]	South Korea	2 weeks	Randomized controlled trial	41	44	The criteria for CAUTIs were fever above 38°C​​​​​​, suprapubic tenderness, and costovertebral angle pain or tenderness; urine culture ≥10^5^ CFU/mL; radical cystectomy and orthotopic neobladder patients
Ackam et al., 2019 [9]	Turkey	Short-term (duration not mentioned)	Randomized controlled trial	28	26	Patients admitted to the ICU with expectant catheterization
Stensballe et al., 2007 [11]	Denmark	<2 weeks (>90% of patients in each group)	Randomized, double-blind controlled trial	77	77	Trauma patients requiring temporary urethral catheterization
Pickard et al., 2012 [12]	United Kingdom	<2 weeks	Randomized controlled trial	Cohort 1: 2,153; Cohort 2: 2,097 Cohort 3: 2,144	N/A	Adults requiring temporary urethral catheterization (one to 14 days)
Bonfill et al., 2017 [13]	Spain	>7 days (median 12 months)	Randomized open-label multicentre clinical trial	243	246	Spinal cord injury patients who needed an indwelling urinary catheter as a method of bladder drainage for at least seven days
Stenzelius et al., 2011 [14]	Sweden	1-3 days	Randomized controlled trial	222	217	Adult patients undergoing elective orthopedic surgery

**Table 3:** Microbiological culture, intervention, urinary tract infection incidence, findings, limitations, and outcome of therapy - indicates no statistical improvement in patient outcome after the intervention; + indicates statistical improvement in patient outcome after the intervention; *bacteriuria instead of CAUTI was determined WBC: white blood cell; CAUTI: catheter-associated urinary tract infection; CFU: colony-forming units; PFTE: polytetrafluoroethylene; UTI: urinary tract infection

Author, year	Microbiological culture: organisms >10 WBC/mm	Intervention	Urinary tract infection incidence: CFU > 10^5^/ml	Main findings	Potential limitations	Outcome of therapy +/-
Tae et al., 2022 [8]	Cohort: 60.89% (significant bacterial colonies) *Enterococcus faecalis*: 19.5% and *Pseudomonas aeruginosa*: 9.75%; Control: 86.36% (significant bacterial colonies) *Enterococcus faecalis*: 25% and *Pseudomonas aeruginosa*: 9.1%	Medicated Foley (silicone and zinc oxide polymer), silicone-coated Foley catheter	Medicated Foley: 21.95%, standard Foley: 27.27%	The incidence of CAUTIs was lower in the case group: nine (21.95%) and 12 (27.27%) patients in the case and control groups, respectively (p = 0.377). No statistically significant difference between the groups was observed.	Size of the study population	-
Ackam et al.,2019 [9]	Cohort *Escherichia coli: *14.3% ; Control* Escherichia coli: *26.9%	Silver-coated silicone Foley, standard silicone Foley catheter	Silver-coated silicone Foley: 46.4%, standard silicone Foley: 46.2%	No difference was found between the use of silver-coated and non-silver-coated standard silicone Foley catheters.	Size of the study population	-
Stensballe et al., 2007 [11]*	Cohort bacteriuria: 9.1%; Control bacteriuria: 24.7%	Nitrofurazone-impregnated catheter, standard silicone Foley catheter	Not determined	Reduction in bacteriuria was noted in the medicated catheter group.	Lack of standardization of patients	+
Pickard et al., 2012 [12]	Cohort 1 pyuria: 26.7%; Cohort 2 pyuria: 27.8%; Cohort 3 pyuria: 27.1%	Cohort 1: nitrofurazone-impregnated catheter; Cohort 2: silver alloy catheter; Cohort 3: PTFE	Cohort 1 UTI 4.1%; Cohort 2 UTI 4.9%, Cohort 3: UTI 4.1%	Reduction in CAUTI was uncertain.	No control group present	-
Bonfill et al., 2017 [13]	Cohort 1 patient developed shock due to *Proteus mirabilis*; Control 2 patients developed shock, one due to *Escherichia coli* and the other due to *Proteus mirabilis*	Silver-coated silicone Foley catheter, standard silicone Foley catheter	Silver-coated silicone Foley catheter: 7.41%; standard silicone Foley catheter: 7.7%	No extra benefit against CAUTIs was noted with a medicated catheter.	Unblinded	-
Stenzelius et al., 2011 [14]*	Medicated catheter bacteriuria: 1.5%; Non-medicated catheter bacteriuria: 5.5%	Noble metal alloy-coated latex catheter, silicone catheter	Not determined	Reduction in bacteriuria was noted in the medicated catheter group.	CAUTI was not determined	+

Discussion

The systematic review revealed that very little information is present on CAUTIs in long-term care facilities and nursing homes as opposed to hospital-acquired infections and acute cases. The majority of patients who suffer from asymptomatic CAUTIs in long-term care facilities have indwelling urinary catheters. These patients should not be treated with antimicrobials unless pregnant or invasive urological interventions are planned [6]. The use of indwelling urinary catheters is a necessity, and thus methods or devices to reduce such CAUTIs were compared in this systematic review. A multitude of studies comparing various medicated and non-medicated indwelling urinary catheters were evaluated. Ultimately, the final outcome is that medicated indwelling urinary catheters don’t confer any added advantage or protection against CAUTIs when compared to the standard Foley catheter. The standard Foley catheter, thus, also has a superior cost-benefit ratio as compared to the more costly medicated catheters [7].

Tae et al. (2022) conducted an RCT among 85 patients who underwent radical cystectomy with an orthotopic neobladder, with 41 intervention patients and 44 control group patients. The inclusion criteria were as follows: The criteria for CAUTIs were fever above 38°C, suprapubic tenderness, costovertebral angle pain or tenderness, and urine culture with ≥105 colony-forming units/mL. The incidence of CAUTIs two weeks after radical cystectomy was found to be not statistically significant in the case group with nine (21.95%) patients as compared to 12 (27.27%) patients in the control group. The intervention group received catheterization with a 20 Fr Foley catheter with silicone and zinc oxide polymers that prevent biofilm formation, and the control group was catheterized with the standard 20 Fr silicone-coated Foley catheter. There was, however, no statistically significant difference in CAUTIs between patients receiving medicated and non-medicated catheters. The most prevalent organisms isolated in the urine were *Enterococcus faecalis* (19.5%) and *Pseudomonas aeruginosa* (9.75%) in the study cohort and *Enterococcus faecalis* (25%) and *Pseudomonas aeruginosa* (9.1%) in the control group, respectively [8].

Ackam et al. (2019) performed a randomized double-blind clinical trial in Turkey comparing silver-coated silicone Foley and standard silicone Foley catheters. The study included patients admitted to the ICU with expectant catheterization. The study outcome revealed that no significant difference in the incidence of bacteriuria and CAUTIs was found between the use of silver-coated and non-silver-coated standard silicone Foley catheters. *Escherichia coli* were the most commonly isolated bacteria in both the cohort and control groups [9]. These findings both align with and further bolster the findings of Tae et al. (2022) [8].

A similar comparative multicentre study conducted by Lee et al. in 2014 on adults requiring catheterization for more than 24 consecutive hours with 92 intervention patients and 85 control group patients had similar outcomes to both the studies performed by Ackam et al. (2019) and Tae et al. (2022). The study compared nitrofurazone (a nitrofuran derivative)-impregnated catheters with the standard silicone Foley catheter. The size of the catheter used was 16 Fr. The incidence of CAUTIs in the standard and medicated catheter groups was 22.4% and 15.2%, respectively, but the difference was not statistically significant. However on subgroup analysis, the incidence of CAUTIs in the patients who had a catheter for five to seven days was lower in the study group (13%) as compared to the control group (18.8%), and it was statistically significant. The commonly isolated organisms in both of the groups were *Enterococcus *and *Pseudomonas aeruginosa* [10]. According to the findings reported by Lee et al., the randomized double-blind clinical trial undertaken by Stensballe et al. in 2007 in 212 adult trauma patients was one of two included randomized studies that concluded that a reduction in catheter-associated bacteriuria and funguria (CABF) was noted in the nitrofurazone-medicated catheter cohort as compared to the standard silicone catheter group. The incidence of CABF was 9.1% in the medicated catheter group and 24.7% in the standard Foley catheter group. The catheter sizes used were 12-16 Fr. The major drawback of the study was that CABF was used as a surrogate marker for CAUTI. However, the clinical relevance of CABF is unknown [11]. A multicenter RCT including 24 National Health Service (NHS) hospitals and 6,394 patients in the UK performed by Pickard et al. in 2012 compared the CAUTIs that developed in nitrofurazone-impregnated, silver alloy-coated, and standard polytetrafluoroethylene (PTFE) Foley catheters in patients who had a Foley catheter for one to 14 days. The rate of development of symptomatic UTI within six weeks of randomization was 10.6% in the nitrofurazone group (n = 2,153), 12.5% in the silver alloy group (n = 2,097), and 12.6% in the PTFE group (n = 2,144), which was not statistically significant amongst the groups. The nitrofurazone-impregnated catheters caused more discomfort for the patients during insertion and removal [12]. The findings of Pickard et al. (2012) are further supported by an open-level multicenter RCT performed by Bonfill et al. in 2017 in spinal cord injury patients, which concluded that no additional benefit against CAUTIs was found with the silver alloy-coated Foley catheter (7.72% symptomatic UTI) when compared to the standard silicone/silicone-latex Foley catheter (7.41% symptomatic UTI). The Foley catheters used were 14-18 Fr in size [13]. Stensballe’s findings are supported by a further study conducted by Stenzelius et al., performed in Sweden on the comparison between standard non-coated latex catheters and noble alloy-coated latex catheters, which concluded that only 1.5% of patients catheterized with the medicated catheter reported a UTI as compared to the 5.5% that was noted in non-medicated catheter cases [14]. The major risk factors noted for the development of CAUTIs in different studies were prolonged duration of catheterization, multiple comorbidities, age greater than 50 years, admission into and/or hospitalization in an orthopedic or urology department, urethral trauma, overdistention of the urinary bladder, catheter insertion outside the sterile surgical theatre, diabetes mellitus, and renal impairment with serum creatinine greater than 2 mg/dL [15-19].

## Conclusions

Catheter-associated urinary tract infections are very common among different patient population strata, and a lot of care should be taken to reduce the likelihood thereof. No significant advantage was noted between the use of medicated and non-medicated standard Foley catheters. It is vital that the correct aseptic technique, placement of the catheter, and proper handling of the urobag are ensured to reduce the likelihood of a CAUTI developing.
